# Two new species of the family Nippobodidae (Acari, Oribatida), including a description of the leg-folding process

**DOI:** 10.3897/zookeys.781.27389

**Published:** 2018-08-14

**Authors:** Nestor Fernandez, Pieter Theron, Sergio Leiva

**Affiliations:** 1 National Council of Scientific and Technological Research (CONICET), Evolutive Genetic Laboratory FCEQyN, Misiones National University, Felix de Azara 1552, 6º, 3300 Posadas Misiones, Argentina Misiones National University Misiones Argentina; 2 Research Unit for Environmental Sciences and Management, North-West University, Potchefstroom, 2520, South Africa North-West University Potchefstroom South Africa; 3 National Institute Agricultural Technology (INTA), Experimental Rural Agency, Aimogastam, Argentina National Institute Agricultural Technology, Experimental Rural Agency Aimogasta Argentina

**Keywords:** Leg-folding process, new cuticular structures, Nippobodidae, systematics

## Abstract

*Nippobodespanemorfis***sp. n.** and *Leobodestrypasis***sp. n.** are described by means of optical and Scanning Electron Microscopy (SEM) and compared to other congeners. The leg-folding process is described and illustrated.

*Nippobodespanemorfis***sp. n.** is characterised by interlocking, double hook-shaped, posterior prodorsal condyle and anterior zone humeral apophysis; posterior prodorsal depression present. Tutorium a large lamina defining a pocket-shaped structure; bothridial opening ovoid, situated at the bottom of a U-shaped structure; deep, rounded-ovoid anterior notogastral depression present; ten pairs of notogastral setae; *c* setae looped, dentate, sharply tipped. Marginal setae *h_3_*, *p_3_* on large promontories, followed by deep V-shaped incision; notogaster completely surrounded by circumgastric depression; lateral genital zone with locking structure constituted by longitudinal cuticular elevation, with promontories and a parallel furrow involved in the leg-folding process; genital plate smaller than anal plate.

*Leobodestrypasis***sp. n.** is characterised by: the presence of posterior prodorsal depression and anterior notogastral depression; bridge-shaped anterior prodorsal condyles; heart-shaped frontal prodorsal orifice; ten pairs of notogastral setae; posterior prodorsal condyle and humeral condyle interlocked, forming double hook-like structure; circumgastric furrow surrounding entire notogaster; setae *lp, h_2_, h_1_* situated on shallow medial furrow; notogastral setae *lm*, *lp*, *h_1_*, *h_2_* medially aligned; *p_1_, p_2_, p_3_, h_3_* marginally situated. Legs I-IV, tutorium, pedotectum I, and pedotectum II involved in leg folding which is inferred to be a protection mechanism.

## Introduction

In 1959 Aoki described the new genus *Nippobodes* from material collected by Mr. K. Kaneko in Hiketa-Machi Kagama, south Japan. Aoki compared the genus to *Tetracondyla*, but in the same paper, without further explanation, included the new genus in the family Carabodidae. *Nippobodesinsolitus* Aoki, 1959 was the first species to be described, and in 1961 Aoki gave a diagnosis for a new family, Nippobodidae, incorporating the genus *Nippobodes*. Other species were later added, such as: *Nippobodeslatus* Aoki, 1970; *N.brevisetiger* Aoki, 1981; *N.yuwanensis* Aoki, 1984; *N.monstruosus* Jeleva & Vũ, 1987; *N.tokaraensis* Aoki, 1989; *N.chejuensis* Choi, 1996; and *N.tamlaensis* Choi, 1996.

*Leobodes* Aoki, 1965 was the second genus to be added to the family Nippobodidiae, with *Leobodesmirabilis*, collected in Mae Ngon Luang, Thailand, as type species. Other species were subsequently described: *L.mirabilis* Aoki, 1965; *L.anulatus* Aoki, 1965; *L.lijiangensis* Aoki, 2000; and *L.yinae* Aoki, 2000.

Three species were collected in China and described as new species of *Nippobodes*: *N.flagellifer* Chen & Wang, 2007; *N.peniculatus* Chen & Wang, 2007 and *N.pseudobrevisetiger* Chen & Wang, 2007. In the same paper the authors added two new species of *Leobodes*: *L.carinatus* Chen & Wang, 2007 and *L.praeconcavus* Chen & Wang, 2007, transferring *Nippobodesmonstruosus* Jeleva & Vũ, 1987 to the genus *Leobodes* as: *L.monstruosus* (Jeleva & Vũ, 1987).

Chen and Wang 2007 proposed a revised diagnosis of the family and provided a key of species described worldwide. That study was based on adult stages, making use of optical microscopy and including some digital images. Legs were discussed in a fragmentary fashion but detailed elements were not included in previous studies and the leg-folding process was not mentioned.

More than five years ago, the current authors embarked on a revision of the Carabodidae family. During these studies, we observed a series of similar characters in Carabodidae and Nippobodidae not discussed in previous studies of Nippobodidae. Aspects such as the leg-folding process, discussed by Fernandez et al. with reference to the Carabodidae family (Fernandez et al. 2013a), are also present in Nippobodidae, with some similarities and significant differences.

Difficulties were encountered in our efforts to provide detailed comparisons with previous papers, mainly due to simplified drawings and descriptions. Frontal and lateral views are often lacking, making it difficult to determine if some structures are absent in previously described species, or if they were not mentioned by authors. We explain the leg-folding process by use of illustrations, complementing the study with SEM micrographs, and include a comparison of the two families.

## Materials and methods

Specimens studied by means of light microscopy followed the techniques described by Grandjean 1949 and Krantz and Walter 2009. Specimens studied under SEM, followed the techniques of Alberti and Fernandez 1990a, 1990b; Alberti et al. 1991, 1997, 2007; Fernandez et al. 1991. Equipment used was the same as for previous studies (see Fernandez et al. 2016).

Optical drawings should be considered semi-schematic with regard to cuticular microsculpture and setal shape. The shape of these specimens made it difficult to orientate the material and obtain the same position consecutively. Studies with SEM provided high levels of precision and detailed Figures; another very important aspect was the positioning system, permitting orientation of material with a much higher level of precision, as well as being able to return to an initial position.

Body measurements taken: total length (from tip of rostrum to posterior edge of notogaster); width (widest part of notogaster). Setal measurements taken on three specimens under SEM. Leg chaetotaxy studies used optical microscopy (standard, polarised, and phase contrast) and SEM.

Setal formulae of legs include the number of solenidia (in parentheses); tarsal setal formulae include the famulus (ε). All measurements are given in micrometres (μm).

Morphological terms and abbreviations used are those developed by Grandjean (1928–1974) (cf. Travé and Vachon 1975; Norton and Behan-Pelletier 2009; Aoki 1959; 1961; 1965a; 1965b; 1989; Fernandez et al. 2013; 2013a, b, c; 2014; Chen and Wang 2007. For setal types Evans (1992: 73) and for ornamentation of cuticular surfaces Murley (1951) were used. Additional terms and abbreviations are given below.

**Abbreviations**:

**MNHG** Museum of Natural History, Geneva, Switzerland.

***a.o*** frontal prodorsal orifice

***a.pr.b*** bridge-shaped anterior prodorsal condyles

***la.le*** lateral ledge

***m.f*** medial shallow furrow

***p.pr.co*** posterior prodorsal condyle

## New taxon descriptions

### Nippobodes panemorfis sp. n.

Taxon classificationAnimaliaSarcoptiformesNippobodidae

http://zoobank.org/9A5846B8-B060-4F6C-9314-1F75B751C374

Figures 1–10 , 11–13 , 14–22 , 23–27 , 28–41 , 42–47 , 48–53

#### Etymology.

The specific epithet “panemorfis” is derived from “panemorfi” (πανεμορφη in Greek) meaning beautiful, due to the aesthetic features of the cuticle and setae.

#### Diagnosis

(adult female). *Prodorsum*. Complex shape; triangular in dorsal view with rounded central posterior zone; double hook-shaped, interlocking posterior prodorsal condyle and anterior zone humeral apophysis; rounded rostrum, with groove and large hump; deep, easily discernible round-ovoid prodorsal posterior depression; tutorium strongly curved, large lamina, connected to prodorsal wall, determining a pocket structure. Reticulate-foveate microsculpture on tutorium, pedotectum I, pedotectum II. Polyhedral bothridium situated under zone where humeral part, overlaps with anterior prodorsal zone; bothridial opening ovoid, located at bottom of U-shaped structure. Notogaster: deep, round-ovoid anterior notogastral depression present; ten pairs of setae *c, la, lm, lp, h_1_, h_2_, h_3_, p_1_, p_2_, p_3_*; setae *c* looped, dentate, sharply tipped; marginal setae *h_3_*, *p_3_* on conspicuous promontories, followed by deep v-shaped incision; circumgastric depression completely surrounding notogaster, originating before setae *la*, running between setae *la, h_1_, h_2_* and *h_3_, p_3_, p_2_, p_1_*; setae *1c*, *3c*, *4b* situated marginally; setae *1b* largest; genital opening on elevated zone; lateral genital zone locking structure with, longitudinal cuticular elevation, promontories with parallel furrow; genital plate smaller than anal plate; adanal setae *ad_1_*, *ad_2_* inserted on elevated zone; *ad_3_* setae smallest.

#### Material examined.

**Holotype**: ♀♀ Female. Label details: “Thailande. Khao Yai National Park (nord-est de Bangkok) Khao Khieo au-dessous d’Air Force check point; 1150 m; versant nord, forêt assez sèche; tamisage débris. 28/XI/1985. Leg: D.H. Burckhardt et L. Löbl”. **Paratypes**: Two adult females, same locality and date as Holotype; deposited in Collection of NHMG; preserved in 70 % ethanol. ***Additional material*** studied using SEM: six specimens, not deposited. “Thailande. Khao Yai National Park (nord-est de Bangkok) Khao Khieo au-dessous d’Air Force check point; 1150 m; versant nord, forêt assez sèche; tamisage débris. 28/XI/1985. Leg: D.H. Burckhardt et L. Löbl”

#### Description.

**Figures 1–10.**
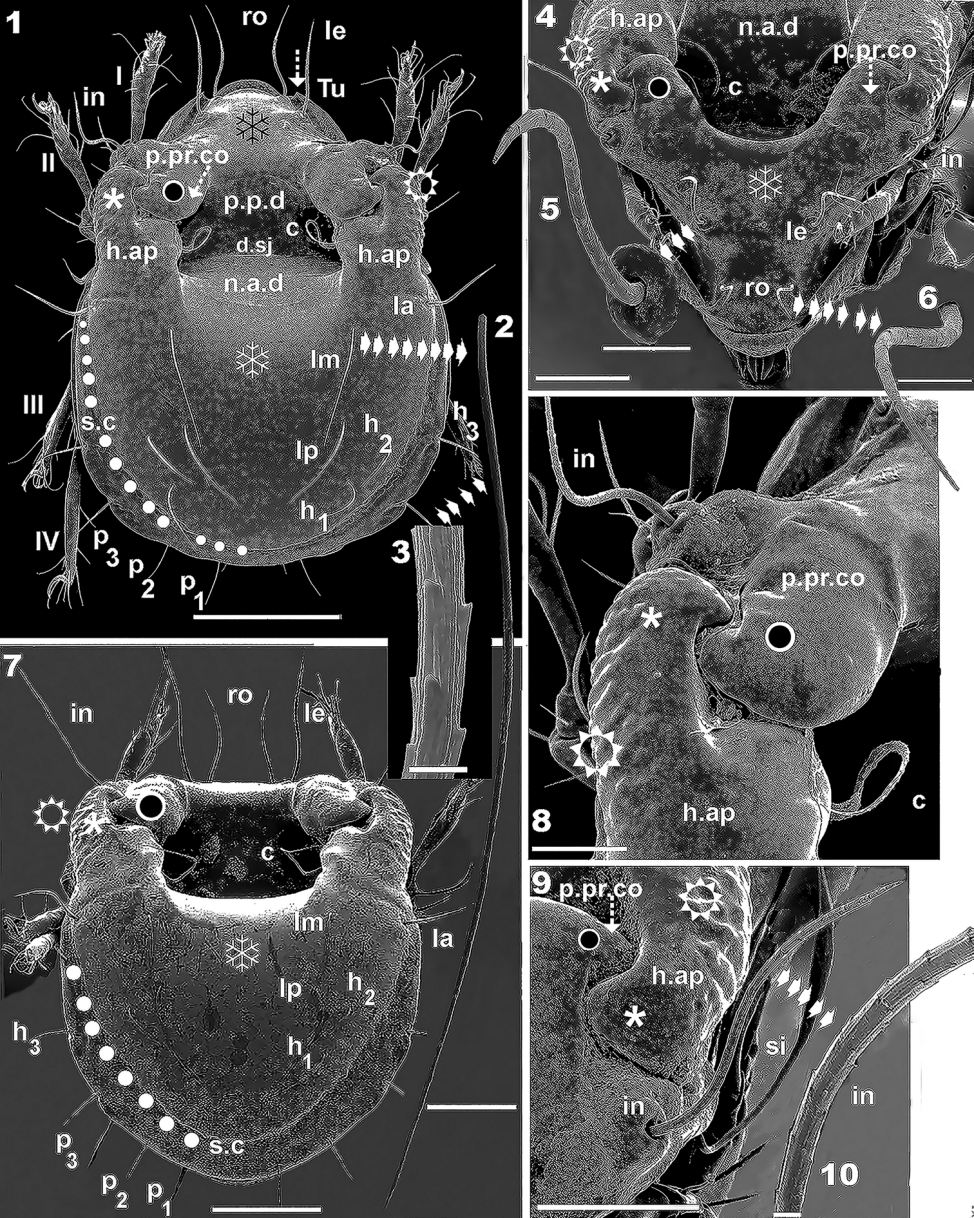
*Nippobodespanemorfis* sp. n. Adult female, SEM. **1** dorsal view **2** detail of notogastral *lm* setae **3** detail of notogastral *lm* setae microsculpture, high magnification **4** frontal view **5** prodorsal lamellar (*le*) setae, detail **6** rostral (*ro*) setae, detail **7** dorsal anteroposterior view **8** prodorsal posterior condyle interlocked with humeral apophysis anterior zone **9** anterior view, humeral apophysis and interlamellar seta **10** interlamellar seta, detail. For abbreviations: see “Material and methods”. Scale bars: 200 μm (**1**); 100 μm (**4, 7**); 50 μm (**5**, **6, 8**); 25 μm (**9**); 17μm (**2**); 3 μm (**10**); 2 μm (**3**).

**Figures 11–13.**
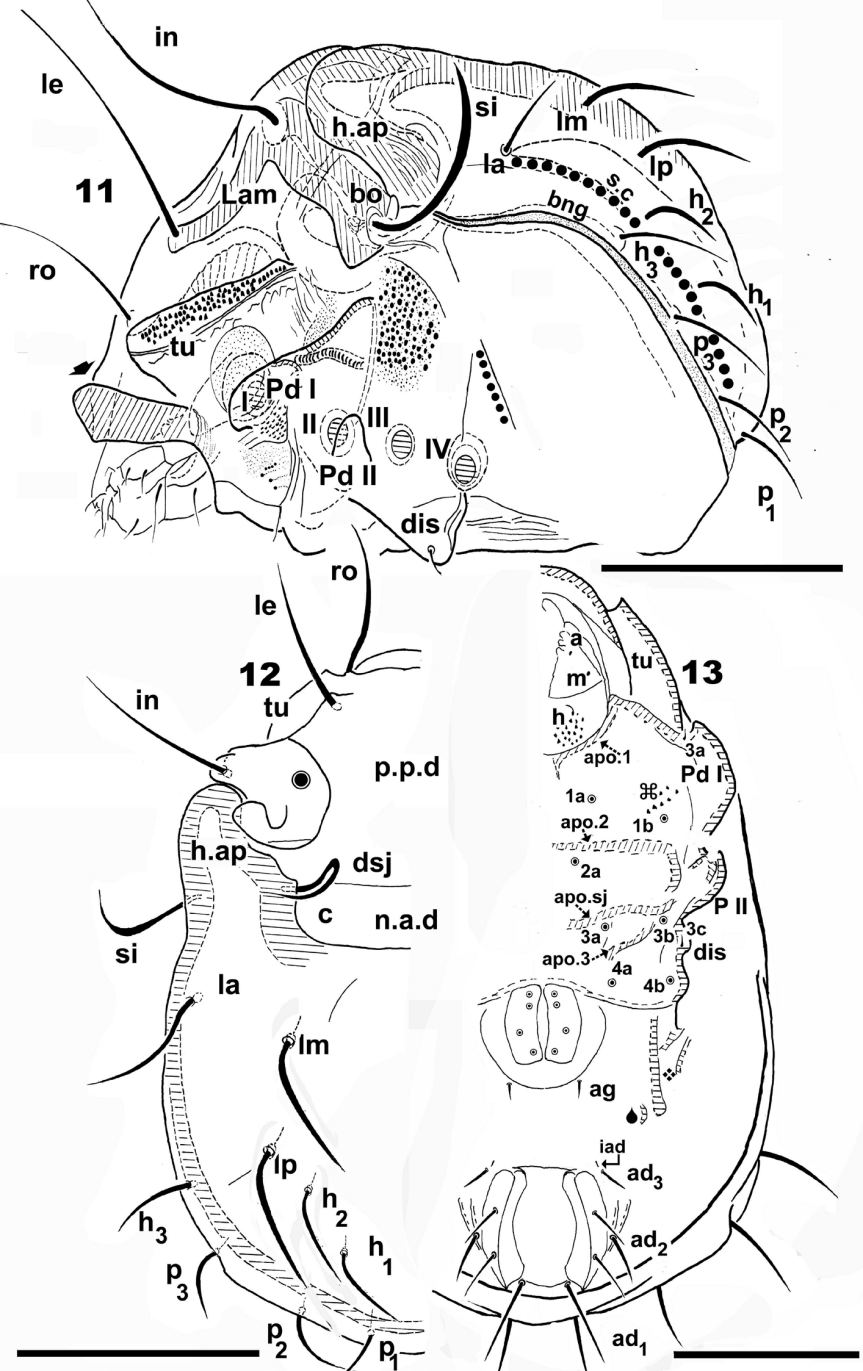
*Nippobodespanemorfis* sp. n. Adult female, Optical observations. **11** lateral view **12** dorsal view **13** ventral view. Scale bars: 200 μm (**11**); 270 μm (**12**); 180 μm (**13**).

*Measurements*. SEM: 597 (542–720) × 368 (332–401) (n = 6). Light microscopy: 610 × 360 (n = 1); all specimens female. *Shape*. Rounded-ovoid (dorsal view) (Figure 1). Elongate oval (lateral view) (Figures 11, 23).

*Colour*. Black, slightly shiny when observed in reflected light; rarely dark brown.

*Cerotegument*. Not observed; small particles, similar to rest of cerotegumental layer on circumgastric depression (*s.c*) lateral zone (Figure 16); the layer may have existed.

**Figures 14–22.**
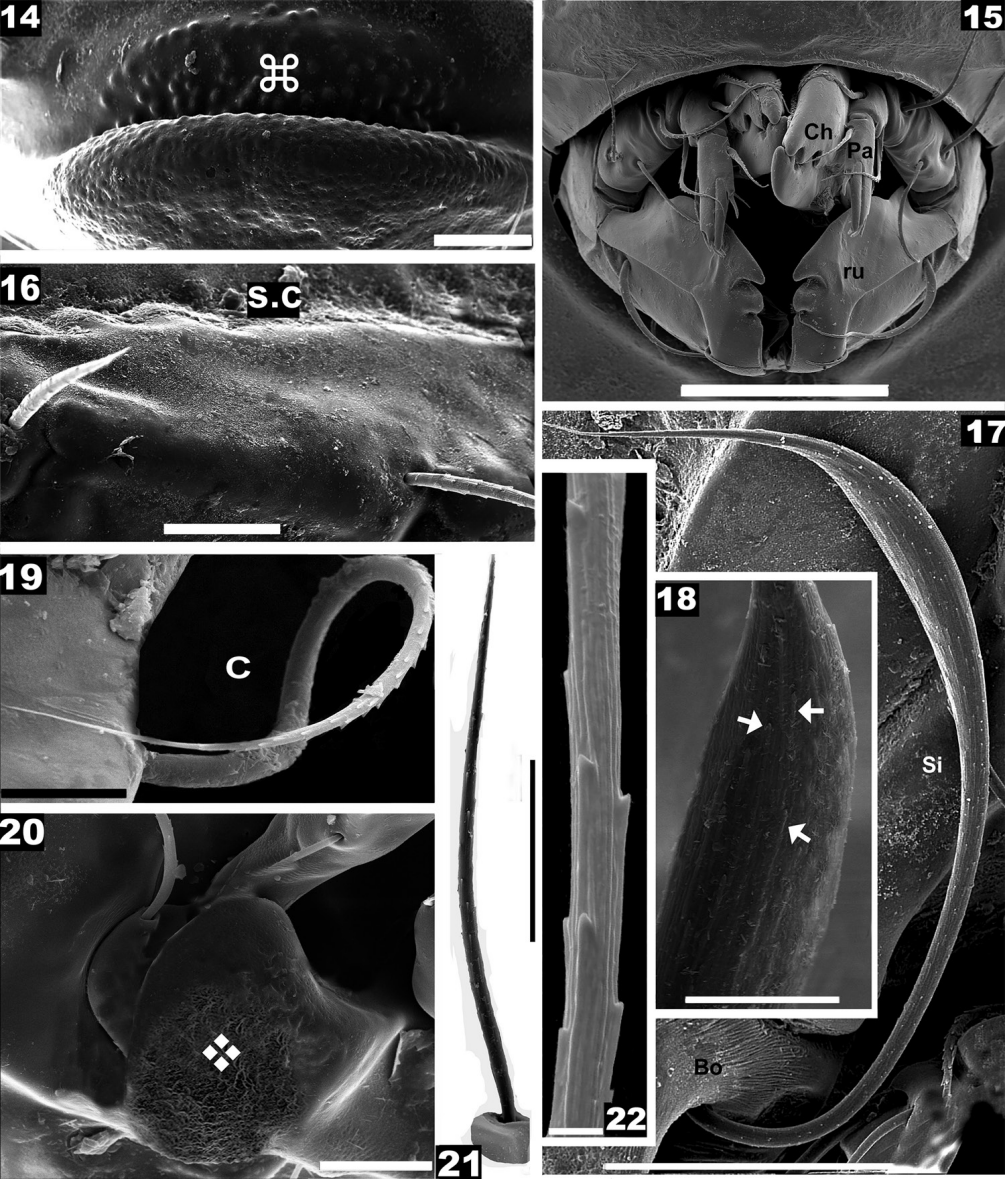
*Nippobodespanemorfis* sp. n. Adult female, SEM observations. **14** rostrum, frontal view **15** subcapitulum, frontal view **16** notogaster, partial lateral view **17** sensillus, lateral view **18** apical zone, sensillus **19** seta *c*, dorsal view **20** pedotectum II, lateral view **21** lamellar seta **22** lamellar seta, detail. Scale bars: 50 μm (**15**, **17**, **20**); 20 μm (**14**, **16**); 10 μm (**18**, **19**); 5 μm (**21**); 2 μm (**22**).

***Integument.*** Microsculpture complex, varying according to body region. *Smooth*: prodorsum (on Figure 4 indicated by ❄); notogaster (on Figures 1, 7 indicated by ❄); on most epimeral surface (on Figures 28, 41 indicated by ❄); genital, anal, aggenital zones. *Tuberculate*: rostum (Figure 14, indicated by ⌘); infracapitulum near setae *h* (Figure 42, indicated by ⌘); epimeral zone between setae *1a*, *1b* (Figure 28, indicated by ⌘).

**Figures 23–27.**
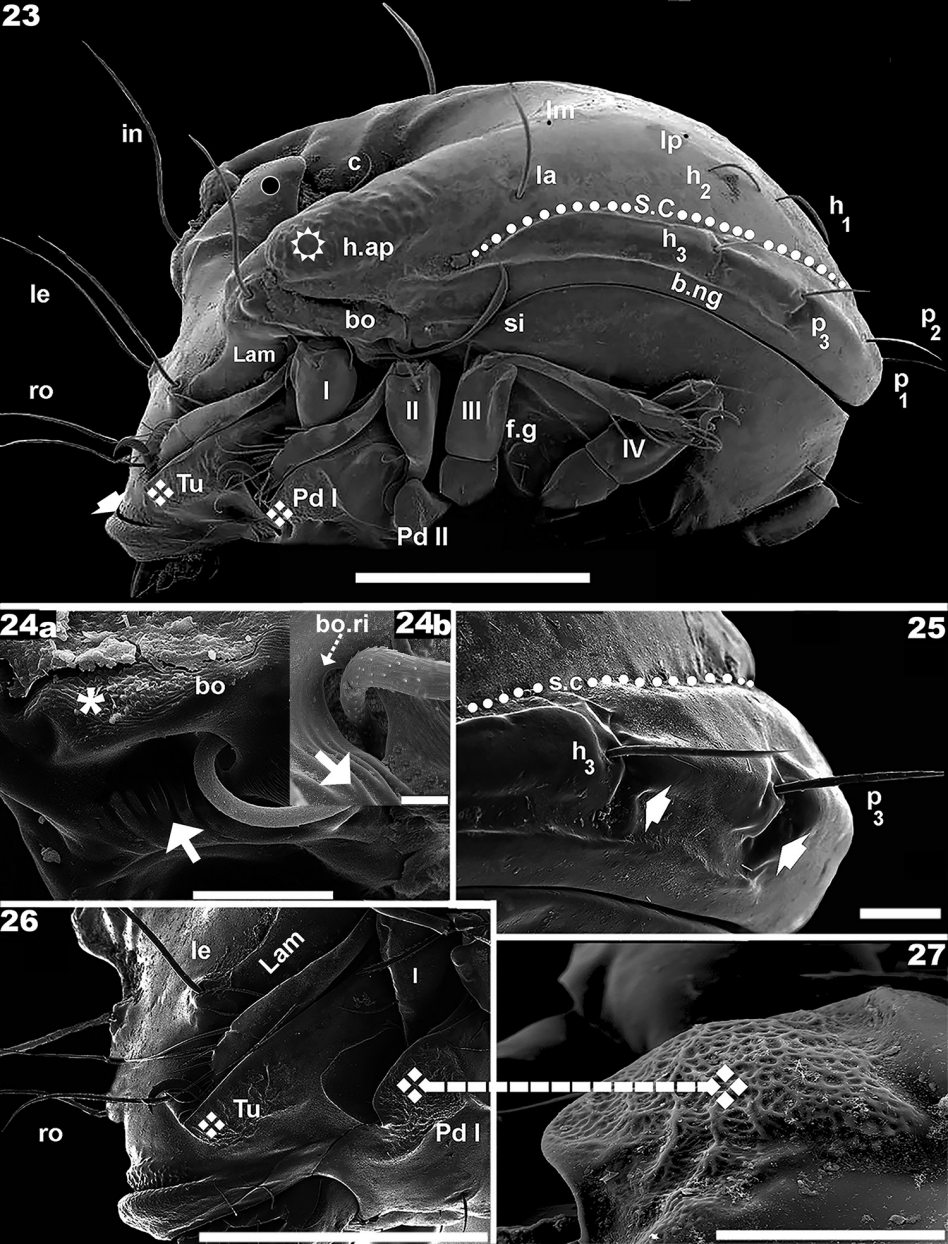
*Nippobodespanemorfis* sp. n. Adult female, SEM observations. **23** lateral view **24a** bothridial zone **24b** bothridial opening **25** lateral view, zone of *h_3_*, *p_3_*, setae **26** prodorsum, lateral view **27** pedotectum I, lateral view. Scale bars: 200 μm (**23**, **26**); 30 μm (**24**, **27**); 20 μm (**25**); 5 μm (**24**).

*Reticulate-foveate*: Tutorium (*Tu*) (on Figure 26 indicated by ❖) ; Pedotectum I (*Pd I*) (on Figures 26, 27 indicated by ❖), Pedotectum II (*Pd II*) (Figure 20, indicated by ❖). *Rugose*: external zone of humeral apophyse (*h.ap*). (Figures 1, 4, 7, 8, 9, 23, indicated by ⛭). *Favulariate*: bothridial zone (Figure 24a, indicated by ✱). *Sulcate*: zone of bothridial opening (Figure 24a, b indicated by ➡). *Punctate*: Discidium (*dis*) (Figure 30 indicated by ➧); epimeral zone surrounding setal insertion (Figures 30, 43, indicated by ➧).

***Prodorsum.*** Complex shape: in dorsal view, more or less triangular with central posterior zone rounded (Figures 1, 4); lateral posterior zone with interlocking double hook-shaped posterior prodorsal condyle (*p.pr.co*) (indicated by ◙) and anterior zone humeral apophysis (*h.ap*) (Figures 8, 9); lateral view: triangular; with complex lateral posterior zone, with double hook and sigmoid lamellae (Figure 23).

Rostrum round, with a conspicuous groove parallel to margin (Figure 23 indicated by ➧) large hump visible in front of groove (Figures 14).

Setae: sigmoid, with small dentitions and thin parallel longitudinal ridges: Rostral (*ro*), interlamellar (*in*), lamellar (*le*) setae (Figures 1, 4, 5, 6, 9, 10, 21, 22): length: 123 (106–141) (n = 12); setae *le*, *in* , inserted each on large tubercle, *setae ro* inserted on small tubercle (Figures 1, 4, 9, 26); setae *ro* situated behind region of tuberculate microsculpture, marginally to depression created by *Tu* and lateral prodorsal wall (Figures 4, 26); *le* setae inserted on anterior end of lamellar zone (Figure 26), situated posteriorly and to the exterior of *ro* setal insertion alignment (Figures 1, 4, 7); setae *in* situated behind and externally to *le* setal insertion level (Figures 1, 4, 7), inserted near the double hook (Figures 8, 9); deep, rounded-ovoid prodorsal posterior depression (*p.p.d*) clearly discernible between dorsosejugal furrow (*d.sj*) and notogastral anterior depression (*n.a.d*) (Figure 1).

Lamellae (*Lam*) clearly visible in lateral view (Figures 11, 23) (see Lateral Region). Bothridium (*bo*) (Figures 23, 24) situated under double hook zone (See: Lateral view). Sensillus (*si*) (Figures 17, 18) sickle-shaped, strongly curved, directing upward with long stalk followed by a swollen zone, plentifully scattered with small asperities and with small barbs (Figure 18 indicated by ➡); long sharp apical tip; length 152 (149–160) (n = 6). *Tu* well developed, sharply tipped; lacking free extremity, welded to lateral prodorsal wall, determining pocket-like structure (Figures 4, 26). Pocket structure conceals leg I when leg-folding process is activated (See: Leg-Folding Process).

***Interlocking double hook.*** The interlocking double-hook zone is complex, formed by *p.pr.co* and anterior zone *h.ap* (Figures 1, 4, 7, 8, 9, 23); where *h.ap* situated externally (indicated by ✱), grips on to *p.pr.co* (indicated by ◙) on the interior. Cuticular surface of *p.pr.co* smooth with some irregular depressions (Figure 8); cuticular surface of *h.ap* rugose externally (Figures 1, 4, 7, 8, 9 indicated by ⛭), internally smooth.

***Notogaster.*** Dorsal view, notogaster polyhedral-rectangular shape (Figure 9); *d.sj*) convex, clearly delimited (Figure 1). Deep, round-ovoid *n.a.d* present, extending posteriorly from *d.sj*.

Ten pairs of setae *c, la, lm, lp, h_1_, h_2_, h_3_, p_1_, p_2_, p_3_*; setae *c* situated on lateral margin of *n.a.d* (Figures 1, 4, 7); setae *c*: *looped, dentate, sharply tipped* (Figure 19). Length: 150 (144–175); *la, lm, lp, h_1_, h_2_, h_3_, p_1_, p_2_, p_3_* (Figures 1, 2, 16, 23, 25): *simple, small dentitions, with parallel longitudinal ridges, sharply tipped* (Figure 3); four pairs situated laterally: *h_3_, p_1_, p_2_, p_3_*; three pairs (*la*, *h_2_*, *h_1_*) situated internally to *s.c*; two pairs (*lm*, *lp*) situated internally to *la*, *h_2_* (Figures 1, 7, 23). Setae *h_3_*, *p_3_* inserted on conspicuous promontories (Figure 1, 23, 25, 42); deep v shaped incision observed behind each seta (Figure, 25, indicated by ➧), determining a scalloped notogastral margin in this region (Figure 1). Setal lengths: *la* 102 (97–112); *lm* 134 (127–142); *lp* 125 (118–132); *h_1_, h_2_* 70 (68–76); *h_3_* 91 (86–98); *p_3_* 59 (56–64); *p_2_, p_1_* 43 (41–47); *s.c* completely surrounding notogaster, originating slightly in front of setae *la*, running between setae *la, h_1_, h_2_* and *h_3_, p_3_, p_2_, p_1_* (Figures 1, 7 trajectory indicated by ●).

***Posterior notogastral view*** (Figure 7). Deep ovoid *p.p.d* as well as *n.a.d* clearly visible; setae *c* situated on paraxial zone of *h.ap*.

Trajectory of *s.c* indicated by ●; externally to *s.c*, flat surface of notogaster extending from *s.c* to notogastral margin; scalloped zone (behind setae *h_3_*, *p_3_*), some distance from *s.c*, not interrupting its trajectory.

***Lateral region.****Tu* strong, large lamina, together with prodorsal wall and lamellae determining a pocket structure; anterior *Tu* ending in sharp angle, with interior part welded to prodorsal wall (Figure 1 indicated by ⇣); behind *le* setal insertion level, pocket structure internally delimited by the *Lam* (Figures 23, 26) (See Leg-folding process). *Pd I*, prominent extended lamina; (Figures 26, 27). *Pd II*, small lamina, rounded apex (Figures 20, 23); *Tu*, *Pd I*, *Pd II* with reticulate-foveate cuticular microsculpture (Figures 20, 26, 27 indicated by ❖).

Complex, polyhedral *bo* situated under the zone where *h.ap* overlaps the anterior prodorsal zone (Figure 23). Bothridial opening observed at the bottom of a long U-shaped structure (Figures 24a, 24b), with sulcate microsculpture on inferior zone (Figure 24a, 24b indicated by ➡); smooth bothridial ring (*bo.ri*) surrounding ovoid botridial opening; *Lam* sigmoid, lacking sharp cuspis (Figures 23, 26); setae *le* inserted on promontories at *Lam* apical zone; *s.c* clearly visible, originating in zone anterior to *la* setal insertion level (Figure 23, trajectory indicated by ●); v-shaped incision observed behind *h_3_*, *p_3_* setae (Figure 25, indicated by ➧); *b.ng* convex (Figure 23).

***Ventral region.*** Epimeral chaetotaxy 3–1–3–2 (Figures 13, 42); setae *1c*, *3c*, *4b* situated marginally (Figure 30); setae *1b* largest (Figure 41); epimeral borders easily observed; *bo.2*, *b.sj* traversing medial plane; *bo.3* small; apodemes *apo.1*, *apo.2*, *apo.dj*, *apo.3* clearly visible (Figure 13); small setae, many small barbs (Figure 30); length: 9 (5–18).

**Figures 28–41.**
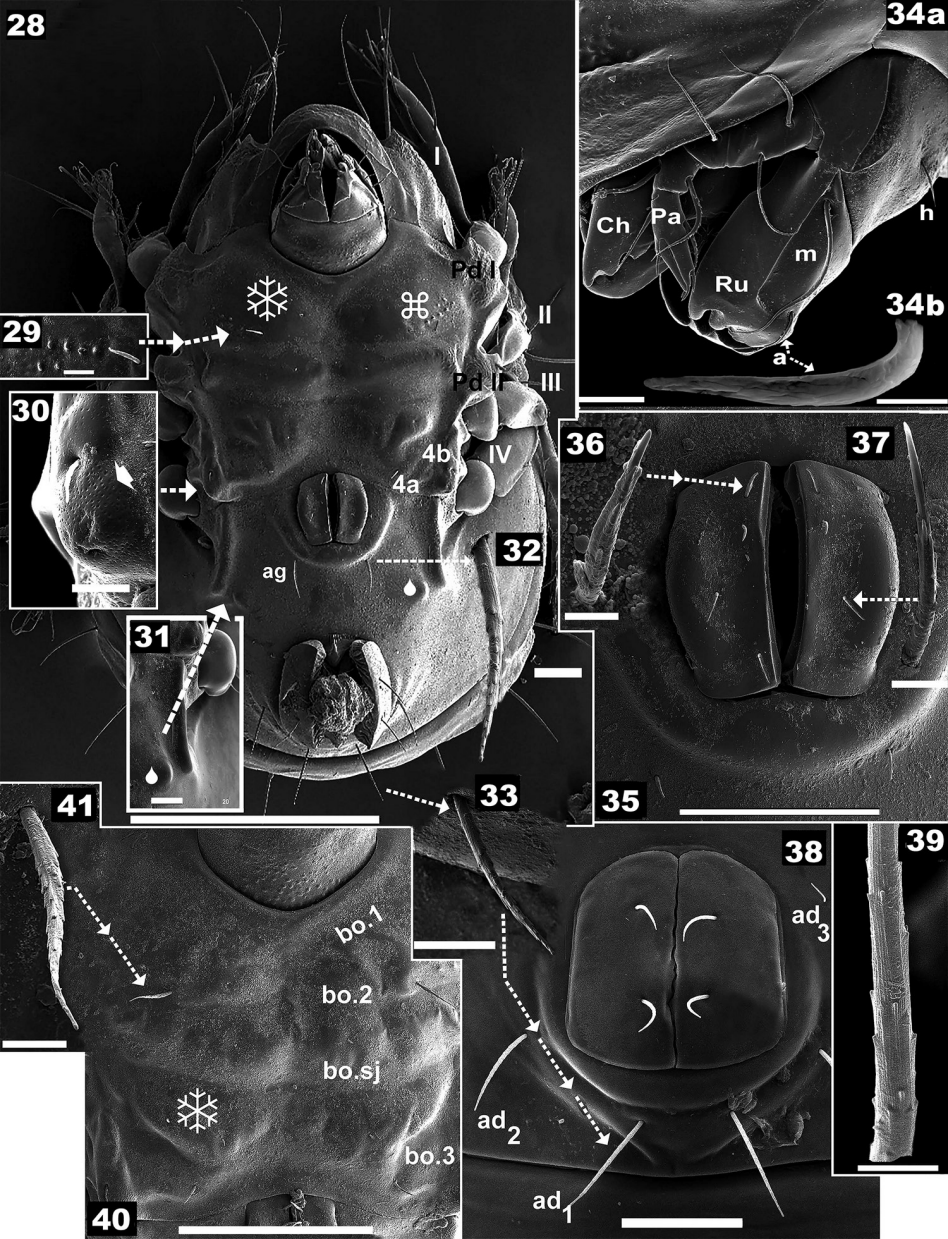
*Nippobodespanemorfis* sp. n. Adult female, SEM observations. **28** ventral view **29** tuberculate zone **30** discidium with epimeral seta *3c***31** ridge associated with leg folding **32** aggenital setae **33** adanal seta **34a** subcapitulum **34b** subcapitular setae *a***35** genital zone **36** genital seta type 1 **37** genital seta type 2 **38** anal zone **39** ornamentation of anal setae **40** epimeral zone **41** epimeral seta *1a*. Scale bars: 200 μm (**28**); 50 μm (**35**, **38**, **40**); 20 μm (**31**, **34**); 10 μm (**29**, **30**, **33**); 5 μm (**41**); 3 μm (**34**); 2 μm (**32**, **36**, **37**, **39**).

**Figures 42–47.**
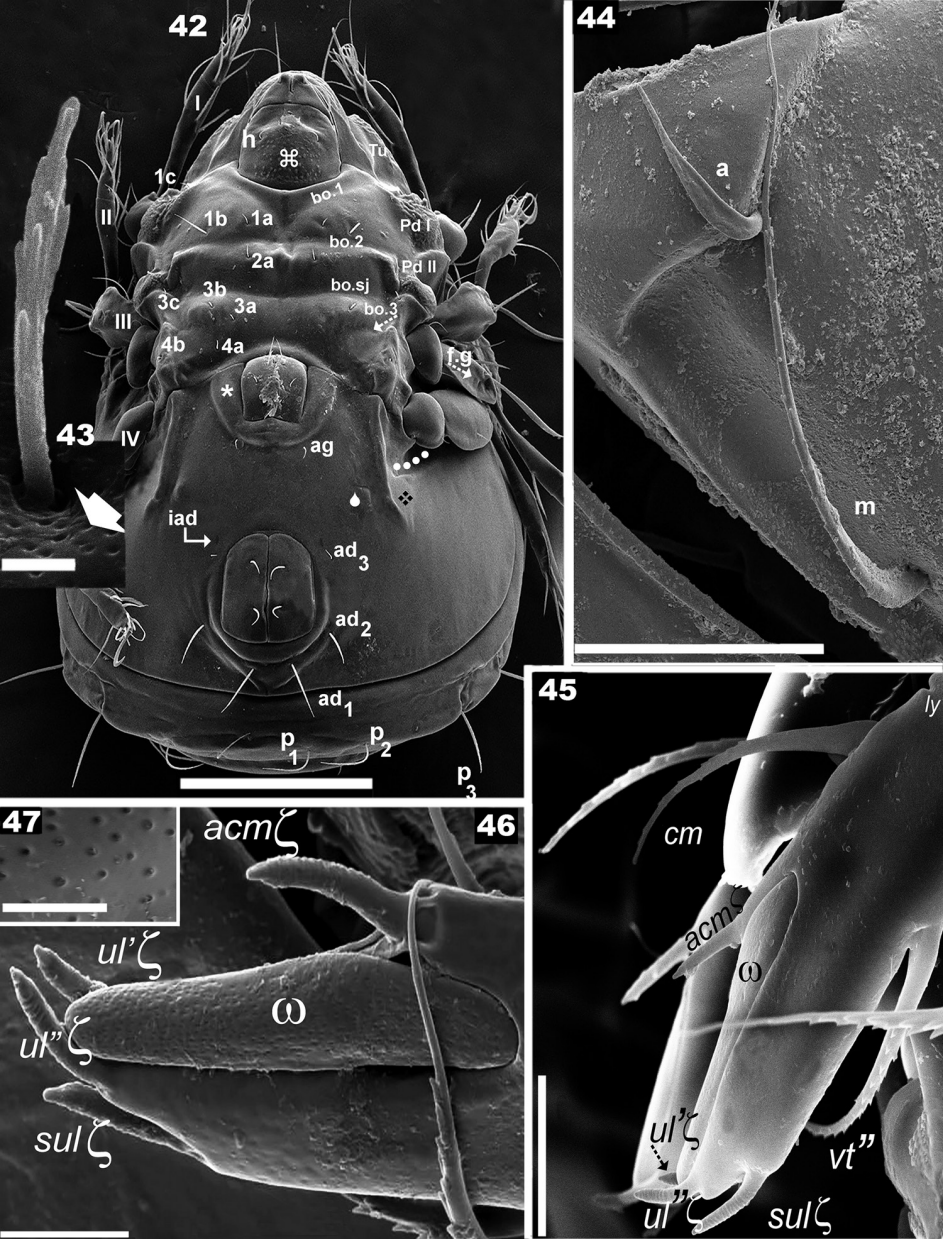
*Nippobodespanemorfis* sp. n. Adult female, SEM. **42** ventral view **43** epimeral seta *2a***44** subcapitulum anterior zone **45** palp, lateral view **46** palp, anterior zone **47** porose area, detail w solenidium. Scale bars: **42** 200 μm; **44** 20 μm; **45** 10 μm; 5 μm (**46**); 2 μm (**43**); 0.1 μm (**47**).

Genital plate ovoid, with four pairs of setae (Figure 35, 36, 37); genital setae: with small barbs, variable in shape (Figures 36, 37); length: 5 (4–7); genital opening on elevated zone (Figures 28, 35); setae *ag* in margin of elevated zone (Figures 28, 32, 42); medium sized setae *ag* with small dentitions, sharp tip (Figures 32) *ag*: 20 (8–21). Complex structure involved in leg-folding process (see Locking structure), situated laterally to setae *ag* (Figures 28, 31, 42); constituted by longitudinal cuticular elevation, with parallel furrow and lateral to it a cuticular promontory and opposite, a polyhedral plate (in Figures 28, 42 indicated by 🌢) (see Leg-folding process); genital plate smaller than anal plate (Figures 28, 42). Anal opening with elevated zone posterior to *h_3_* insertion level (well visible in ventral posterior view) (Figure 42); anal plate more or less rectangular with rounded anterior and posterior zones; two pairs of anal setae (Figures 28, 32, 38, 42).

Setae: *an* small dentitions, parallel ridges (Figure 39), length 14 (15–20); three pairs of adanal setae (Figures 28, 33, 38, 42); setae *ad_1_*, *ad_2_* inserted on elevated zone; *ad_3_* setae smallest (Figures 38, 42); adanal setae: medium sized, small dentitions, sharply tipped (Figures 32, 33), length 32 (30–36). Three pairs of subcapitular setae, *h*, *m* and *a*: *h, m* simple, finely barbate (Figures 34a, 44), *a* elongated leaf-shaped, with some narrow, shallow longitudinal furrows (Figure 34b); setae *h* situated in margin of tuberculate zone (Figure 42 indicated by ⌘); setae *m* curving, lengths: *h* 10 (9–12); *m* 30 (28–32); *a* 11 (10–12).

Palp (Figure 34): the first four segments display normal setation (0–2–1–3); tarsus particular, presenting only: *cm* barbate, (*vt*) barbate, w solenidion and eupathid *acmx*, *sulx*, *ul’x*, *ul”x*. Solenidion unusually shaped (Figures 45, 46), with porous surface (Figure 47); eupathid *sulx*, ul’*x*, ul”*x* with an obvious apical perforation (Figure 46).

**Figures 48–53.**
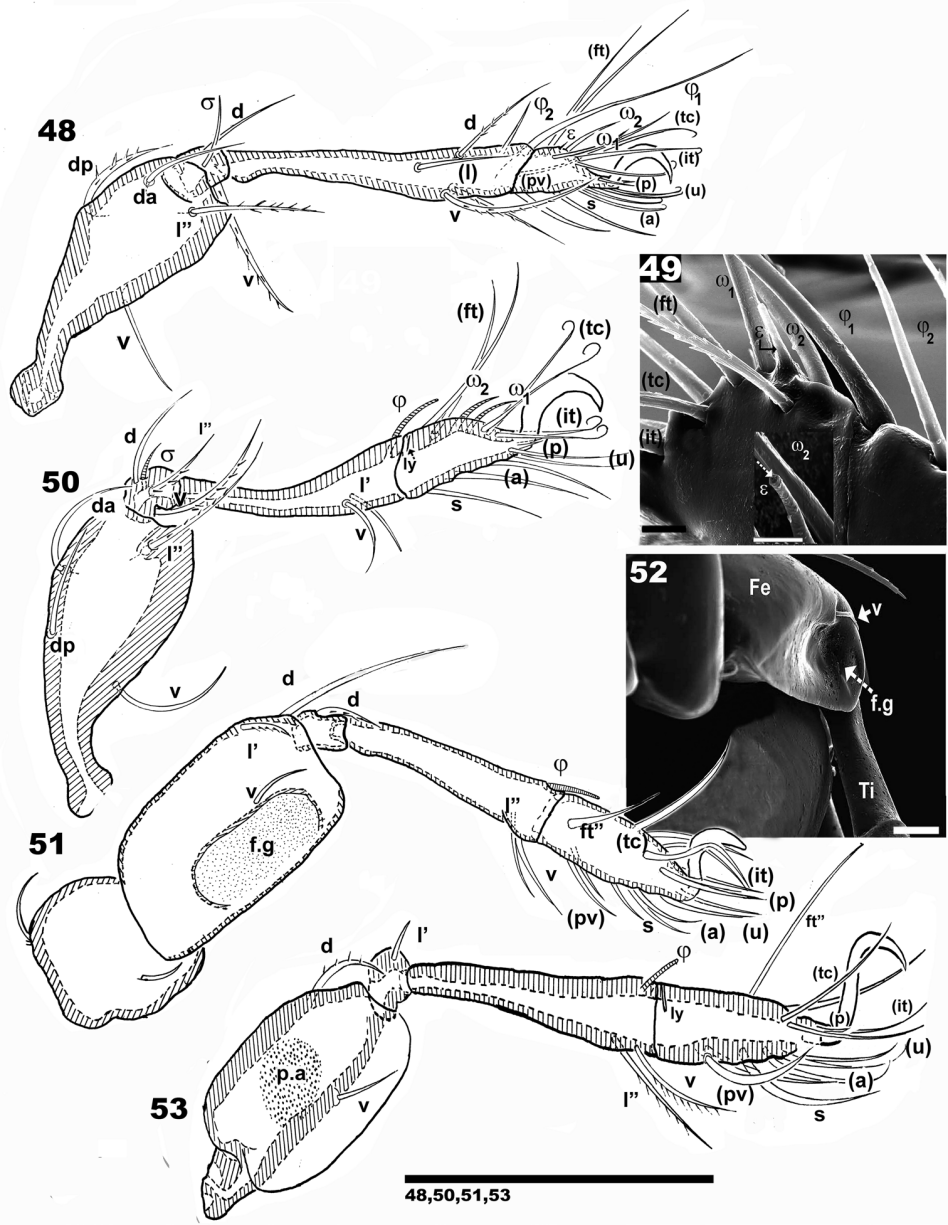
*Nippobodespanemorfis* sp. n. Legs. Adult female, optical observations. **48** leg I antiaxial **49** leg I, detail solenidia and famulus **50** leg II antiaxial **51** leg III, antiaxial **52** femur III ventral, detail of femoral groove **53** leg IV antiaxial. Scale bars: 100 μm (**48**, **50**, **51**, **53**); 15 μm (**49**); 10 μm (**52**).

***Legs.*** See Figures 48–53, Table 1. All legs with very small genu and long tibia. Femur leg IV with large round porose area (Figure 53). Femur III with large femoral groove (*f.g*) (Figures 51, 52) (see Leg-folding process). Setal formulae I (1–4–2–4–16–1) (1–2–2); II (1–4–3–2–15–1) (1–1–2); III (2–3–1–2–15–1) (0–1–0); IV (1–2–1–2–14–1) (0–1–0).

**Table 1.** *Nippobodespanemorfis* sp. n. setae and solenidia.

	Femur	Genu	Tibia	Tarsus	Claw
**Leg I**					
setae	*dp,da,l”,v*	*d,v*	*d,(l), v*	*(ft)*,ε,*(tc),(it),(p),(u),(a),s,(pv)*	1
solenidia		σ	φ*_1,_*φ*_2_*	ω*_1,_*ω*_2_*	
**Leg II**					
setae	*dp,da,l”,v*	*d,v,l*”	*l’v*	*(pv)(ft),(tc),(it),(p),(a),(u),s*,	1
solenidia		σ	φ	ω *_1,_* ω *_2_*	
**Leg III**					
setae	*d,l’,v*	*d*	*l’,v*	*(pv),s,(a),(u),(p),(it),(tc),(ft)*	1
solenidia			φ	-	
**Leg IV**					
setae	*d,v*	*l*’	*l”,v*	*ft”,(tc),(it),(p)(u),(a),s,(pv)*	1
solenidia		-	φ	-	

#### Remarks.

Future ontogenetic studies are necessary in order to confirm nomination of notogastral setae. As only the adult stase was available for study, we used standard, previously used notation (see Morphological terminology). We were unable to locate information on the palp in previous studies. Setae *l*’ of genu II were indicated by Chen and Wang (2007) as bifurcate; however, in our studies only one instance of bifurcate setae *l*’ was observed. Another particularity is the presence of (*it*) on tarsus IV. The femoral groove was observed, though not indicated in any previous study.

### Leobodes trypasis sp. n.

Taxon classificationAnimaliaSarcoptiformesNippobodidae

http://zoobank.org/09F0BA2C-4623-46E3-8275-DFFA740A3966

Figures 54–58 , 59–61 , 62–70 , 71–72 , 73–76

#### Etymology.

The specific epithet “trypasis” is derived from τρύπα in Greek meaning a hole, due to the characteristics of the anterior prodorsum.

#### Diagnosis.

Rostrum ovoid; smooth cuticula with isolated verrucous tubercles; setae *ro* sigmoid; setae *le* curved, directing forward and upward; setae *in* slightly sigmoid, directing forward; deep round-ovoid posterior prodorsal depression; massive posterior prodorsal condyles, posterolaterally located, extending anteromedially to form a curved bridge, interlocking medially in undulate zone. Lateral lamellae, curved ribbon; frontal orifice, heart shaped; translamella curved; tutorium welded to lateral prodorsal wall, determining pocket structure, sharply tipped, but welded to lateral prodorsal wall; sensillus sickle-shaped, strongly curved, upwards; long stalk, swollen middle zone, apically long sharp end. Anterior notogastral depression deep, ovoid-elongate shape; humeral apophysis overlapping posterior prodorsal condyle, extending to the proximity of interlamellar setae; circumgastric depression, surrounding whole entire notogaster; setae *c* hook-shaped; flat smooth surface surrounding laterally whole notogaster; flat, smooth lateral ledge, surrounding entire notogaster; anterior zone, ribbon shaped; genital plate smaller than anal plate.

#### Material examined.

**Holotype**: ♀ Female “VN 12/03c Vietnam. Vinh Phuc Prov. evergreen Forest 1 km SE Tam Dao city. 21°26'49"N, 105° 39'06"E. 13/14/V/2012. Leg. P. Schwendinger & A. Schulz”.

#### Description.

Measurements. SEM: 680 (610–750) × 336 (302–400) (n = 5). Light microscopy: 701 × 341 (n = 1); all specimens female.

**Figures 54–58.**
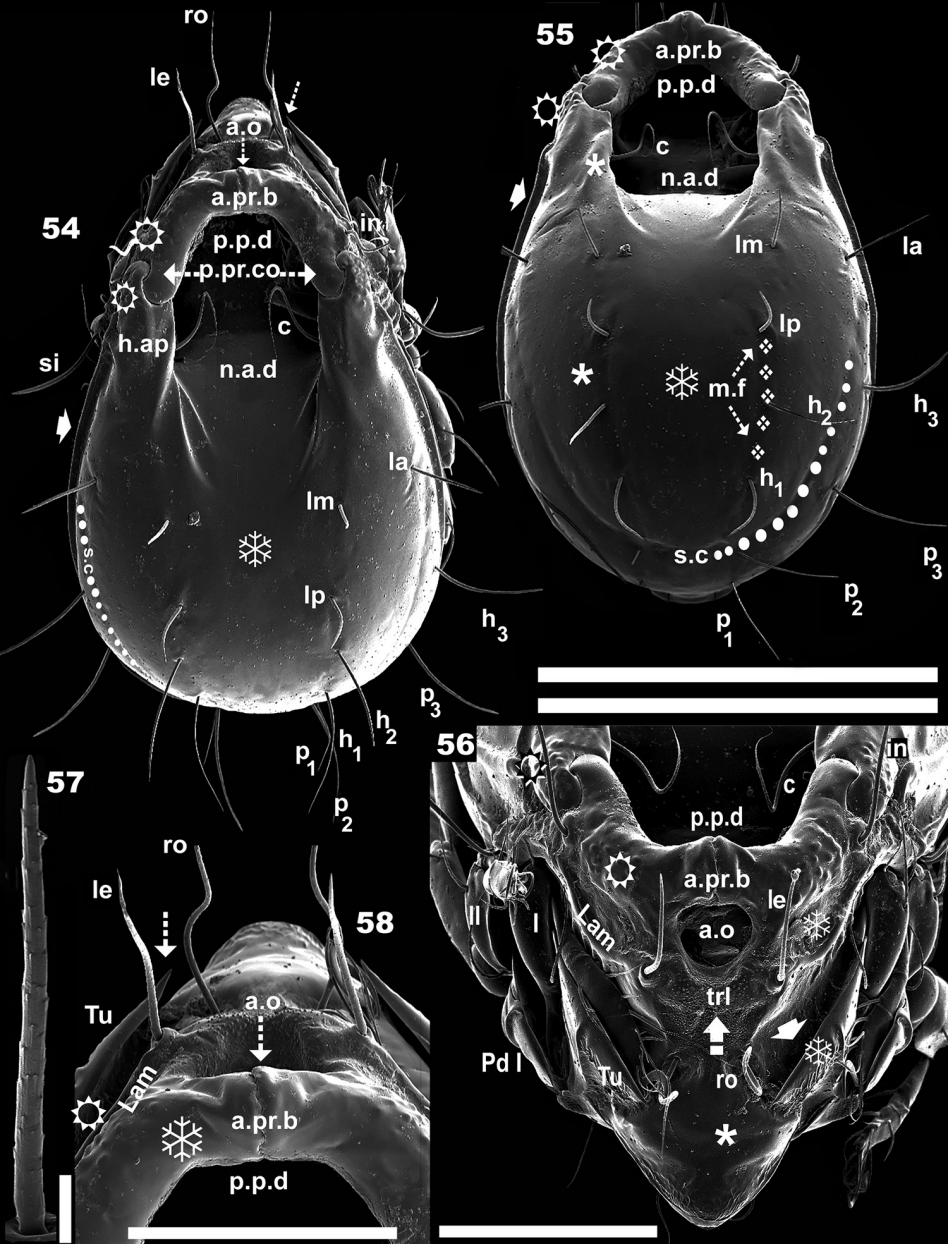
*Leobodestrypasis* sp. n. Adult female, SEM. **54** dorsal view **55** posterior dorsal view **56** prodorsum anterior view **57** notogastral seta *la***58** anterior prodorsal zone. Scale bars: 500 μm (**54**); 300 μm (**55**); 150 μm (**56**); 16 μm (**57**).

Shape. Oval (Figure 54).

Colour. Dark brown to black; slightly shiny when observed in reflected light.

Cerotegument. Not present.

***Integument.*** Microsculpture varying according to body region: *Smooth*: *p.pr.co* interior zone; transversal bridge-shaped structure (*a.pr.b*) (Figures 54, 55, 58); superior *Lam* zone; superior and apical region *Tu* (on Figures 56, 65 indicated by ❄). Notogaster: central zone; marginal zone between *s.c* and *b.ng* (on Figure 62 indicated by ❄); flat lateral ledge (*la.le*) situated immediately above *b.ng* (see below) (on Figures 62, 63 indicated by ❄); epimeral zone (on Figures 71, 72 indicated by ❄); infracapitulum (on Figure 71 indicated by ❄). *Tuberculate* (two types). *Small tubercles*: prodorsal zone below frontal orifice (*a.o*) and between setae *le* (on Figure 56 indicated by ➠); *large verrucous tubercles* (Figure 70): isolated tubercles, dispersed on prodorsum (Figures 56 indicated by ✱), notogaster (Figure 55, indicated by ✱), and ventral zone (Figure 62, indicated by ✱). *Rugose*: external zone *h.ap*, external zone of *pr.co* (Figures 54, 55, 56, 58 indicated by ⛭). *Reticulate-foveate*: basal zone of *Lam*, (Figure 62, indicated by ❖); *Tu* (Figure 62, indicated by ❖); *Pd I* anterior zone. *Sulcate*: area of bothridial opening (Figure 64, indicated by ➡).

**Figures 59–61.**
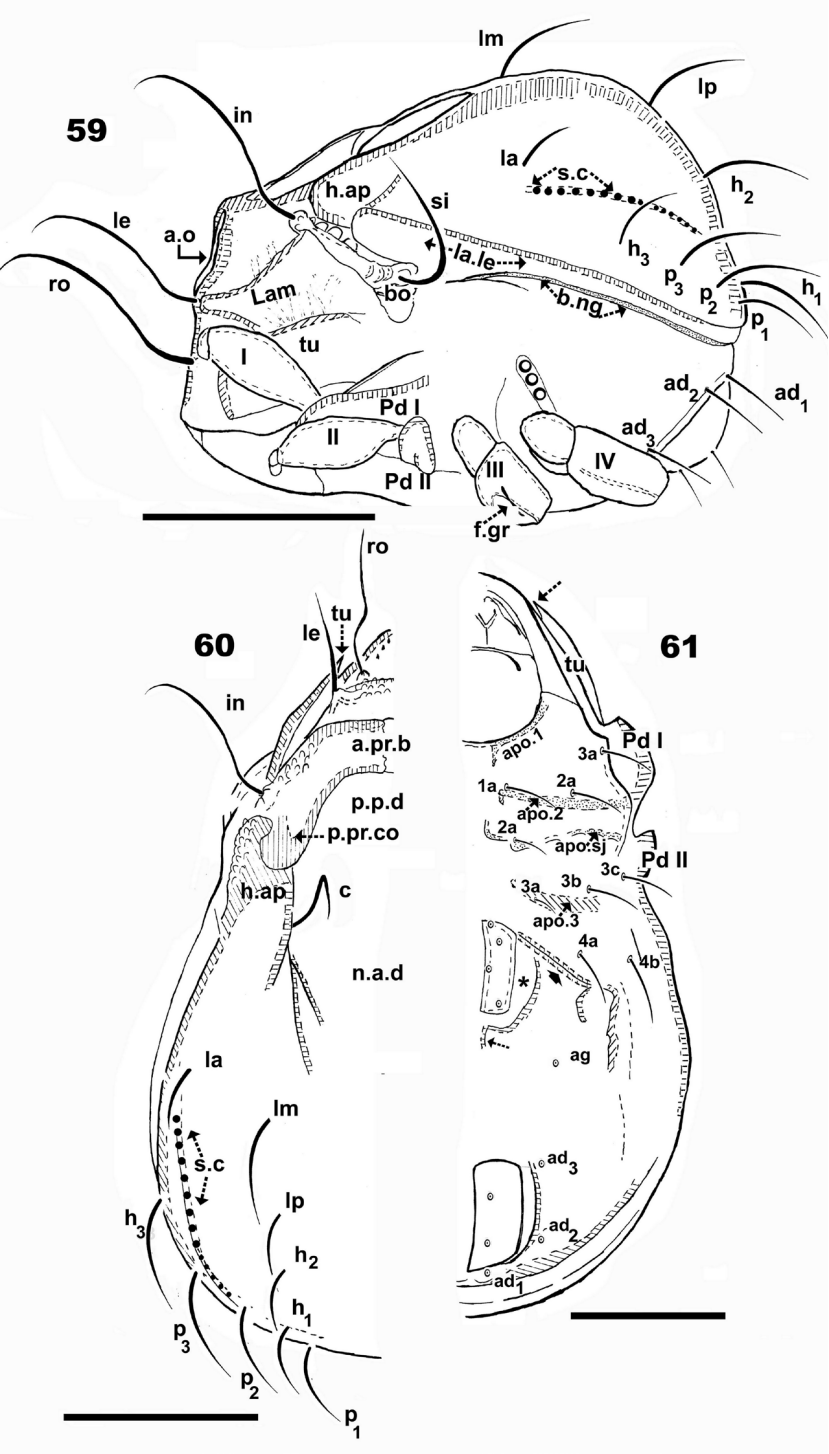
*Leobodestrypasis* sp. n. Adult female, optical observations. **59** lateral view **60** dorsal view **61** ventral view. Scale bars: 280 μm (**59**); 250 μm (**60**); 200 μm (**61**).

**Figures 62–70.**
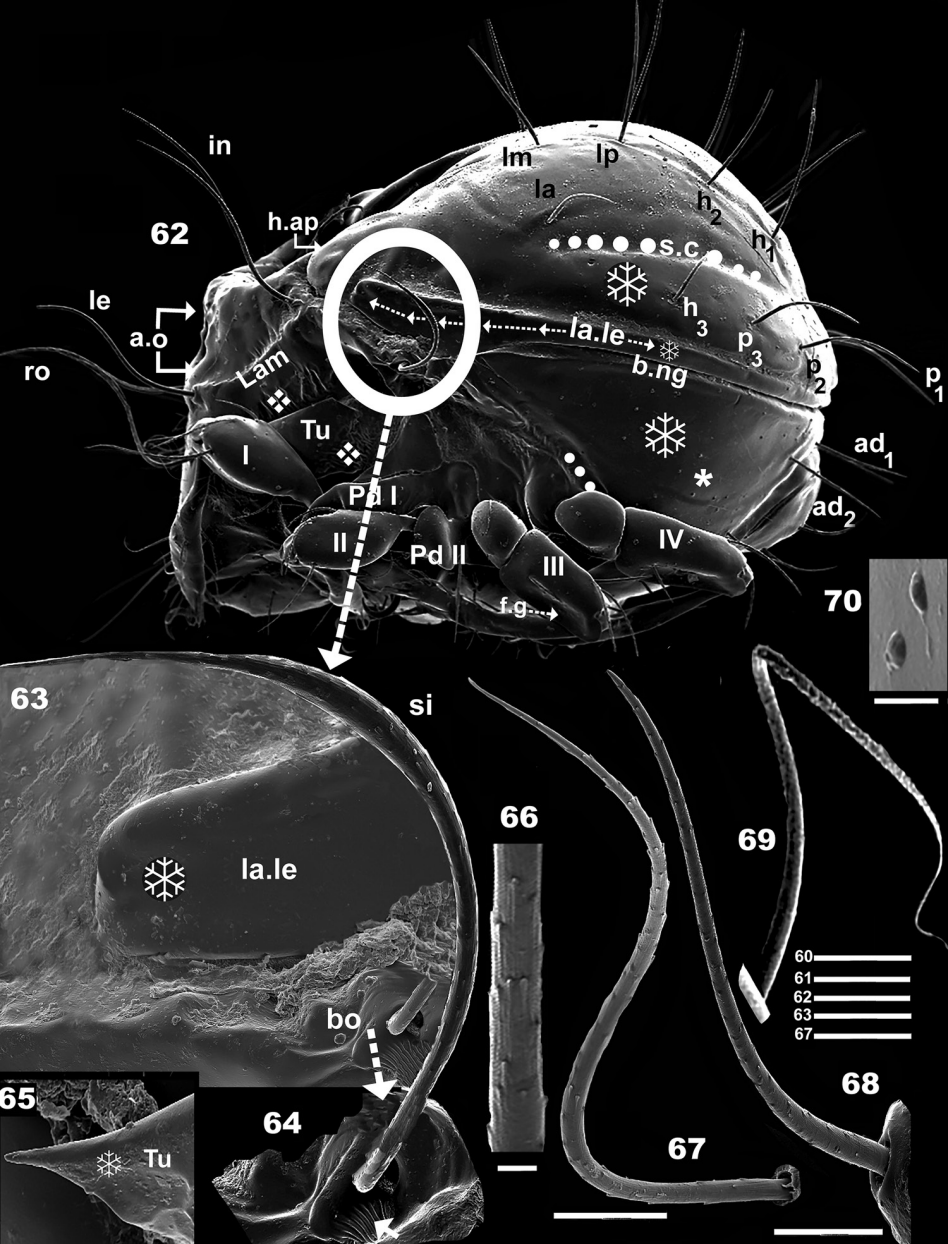
*Leobodestrypasis* sp. n. Adult female, SEM observations. **62** lateral view, (circle indicating the area where *bo*, *si*, *la.le* are situated (see Figures 63, 64) **63** detail, humeral apophysis **64** bothridium, sensillus detail **65** apical zone *Tu***66** detailed microsculture, notogastral setae **67** setae *ro***68** setae *le***69** setae *c***70** large verrucous tubercles. Scale bars: 100 μm (**62**); 32 μm (**69**); 25 μm (**68**); 20 μm (**63**, **64**, **65**, **67**); 2 μm (**66**); 0,7 μm (**70**).

Setation (legs not included). *Simple, smooth*: subcapitular setae *a. Simple, small, dentate, parallel longitudinal ridges* (Figure 66): prodorsal (Figures 67, 68), notogastral (Figure 57, 69), epimeral, subcapitular *m*, *h*; genital, aggenital, adanal, and anal setae.

***Prodorsum.*** More or less triangular in dorsal view, central posterior concave; lateral view: polyhedral (Figure 62); lateral posterior zone with double hook, interlocking posterior prodorsal condyle (*p.pr.co*) and *h.ap* anterior zone (Figures 54, 55). Rostrum ovoid. Smooth cuticula with some isolate verrucous tubercles (Figures 56, 58) on zone delimited by rostrum and *ro* setal insertion level. Setae *ro*, *le*, *in* inserted each on large tubercle; *ro* sigmoid, directing forward (Figure 67), length 150 (142–161) (n = 10); *le* setae curved, directing forward and upward, length: 106 (102–112) (n = 10), situated on apical lamellar zone (Figures 56, 68); setae *in* length: 178 (172–190) (n = 10); slightly sigmoid, directing forward, situated externally, anterior to *h.ap* (which overlaps with posterior prodorsal zone) and posterior to rugose lateral zone of *p.pr.co* (Figures 56); *p.p.d* clearly discernible, deep, round-ovoid in shape (Figure 54, 55, 56, 58). Massive *p.pr.co*, hook-shaped, located posterolaterally; anteromedially curved bridge (*a.pr.b*), interlocking medially in an undulate zone (Figures 54, 55, 56, 58).

Lamellae *(Lam)* clearly visible; lateral longitudinal rib, dorsally concave (Figure 56, 57). Conspicuous heart-shaped *a.o* (Figures 56, 58), located below the *a.pr.b*, and between setae *le*, limited inferiorly by Translamella (*trl*); *trl.* a curved structure, running parallel to and below *a. o* (Figure 56). *Tu* well developed; welded to lateral prodorsal wall, determining a pocket (Figures 56 indicated by ➧); large sharp tip (Figures 54, 58 indicated by ⇣). *Bo* complex (Figures 62, 64), situated under *la.le* (see: Lateral region). *Si* (Figure 64) sickle-shaped, strongly curved, directing upward with long stalk, followed by swollen zone, long sharp apical tip; plentiful small asperities and small barbs on swollen zone (Figure 64); length: 150 (146–161) (n = 12).

***Notogaster.*** Deep, elongate ovoid *n.a.d* present, extending from posterior to more or less half of total notogastral length; medial posterior *n.a.d* zone, open without clearly defined margin; *n.a.d* lateral marginal zone with three lines; more externally: a short, concave line on interior of *h.ap* margin; rectilinear central line; third line lateral *to* posterior margin (Figure 54). Anterior *h.ap* zone overlapping *p.pr.co* (double hook), extending to the proximity of setae *in* (Figures 54, 56); *s.c* completely surrounding the notogaster; originating at level of *la* setal insertion, running internally to setae *h_3_, p_3_, p_2_, p_1_* (Figures 54, 55, 62 trajectory indicated by ●).

Ten pairs of setae: *c, la, lm, lp, h_1_, h_2_, h_3_, p_1_, p_2_, p_3_* (Figures 60, 62); setae *c* hook-shaped (Figure 69), situated on lateral margin of *n.a.d* (Figures 54, 55, 56); four pairs situated marginally: *p_1_, p_2_, p_3,_ h_3_*; four, more or less aligned pairs *lm*, *lp*, *h_2_*, *h_1_*, situated internally (Figure 55); *lp*, *h_2_*,*h_1_* on medial shallow furrow (*m.f*); only clearly discernible in dorsoposterior view (on Figure 55 indicated by ❖); setae *la* situated between *h_3_, p_1_, p_2_, p_3_* and *lm*, *lp*, *h_2_*, *h_1_* (Figures 54, 55) . Setal lengths. *c*: 167 (156–172); *la*: 100 (83–102); *lm*: 75 (72–81); *lp*: 83 (81–89); *h_3_*: 125 (123–131); *h_2_*:145 (93–147); *h_1_*: 154 (101–162); *p_3_*: 125 (100–132); *p_2_*: 145 (116–137); *p_1_*: 73 (71–78).

***Lateral region.*** The tutorium (*Tu*) strong, large lamina, attached to prodorsal wall, determining a pocket structure; terminating anteriorly in long sharp tip (Figure 65); the welded zone of *Tu* is U-shaped, and the claw of leg I is extended outwards during the leg-folding process (Figure 56) (see Leg-folding process).

Lamella (*Lam*) forming conspicuous curved ribbon (Figure 56, 59); running more or less parallel to *Tu* margin; setae *le* situated on promontories on apical zone. *Pd I*: prominent lamina, directing forward, slightly tilted down. *Pd II* a small lamina, rounded apex; on basal zone a small hump directing outwards (Figure 62). The area immediately above *b.ng* is flat, smooth, surrounding the entire notogaster (Figure 62, trajectory indicated by ⇣); this flat surface, forms a prominent *la.le*, parallel to *h.ap* (Figure 62); *la.le* anterior zone, ribbon shaped, (Figure 59, 62, 63); *b.ng* slightly convex (Figure 62).

*Bo* complex: polyhedral, situated below *la.le* (Figure 64); bothridial opening situated at the bottom of a long U-shaped structure; inferior zone with sulcate microsculpture (Figure 64), indicated by ➡); bothridial opening ovoid, surrounded by smooth *bo.ri*; *s.c* clearly visible, originating at level of *la* insertion setal level (Figure 62 indicated by ●).

**Figures 71–72.**
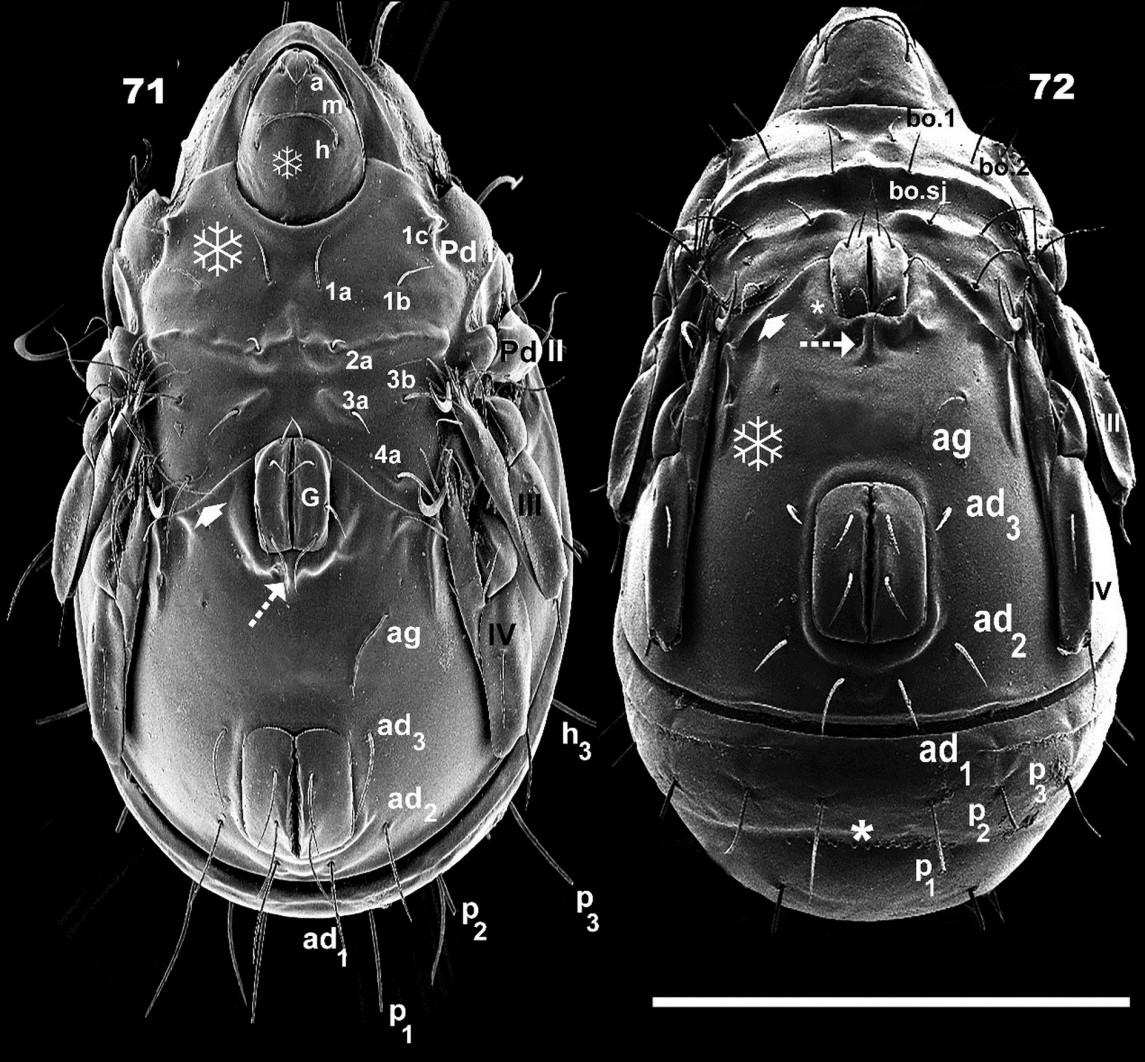
*Leobodestrypasis* sp. n. Adult female, SEM observations. **71** ventral view **72** posterior anterior view. Scale bars: 450 μm (**71**, **72**).

***Ventral zone.*** Epimeral chaetotaxy 3-1-3-2 (Figures 61, 71, 72); setae *1c*, *3c*, *4b* situated marginally; setal lengths: *1a*: 35 (32–38); *1b*: 35 (30–37); *1c*: 29 (25–31); *2a*: 12 (10–15); *3a*: 22.5 (20–25); *3b*: 38 (35–42); *3c*: 18 (16–19); *4a*: 50 (45–52); *4b*: 38 (41–45); epimeral borders clearly visible; *bo.sj* crossing transverse medial plane (Figure 72); *bo.3* small; apodemes *apo.1*, *apo.2*, *apo.dj*, *apo.3* clearly visible (Figure 61). *Pd I*, *Pd II* clearly discernible (Figures 71, 72).

Genital aperture rectangular, anterior margin rounded, four pairs of setae: *g_1_*: 71 (68–73); *g_2_* : 49 (44–52); *g_3_*: 37 (34–43); *g_4_*: 35 (31–38); Elevated ridge surrounding genital opening medially and towards posterior zone (Figure 72, indicated by ✱), in posterior zone a small vertical column present (Figure 71, 72 indicated by ⇣); setae *ag* distanced from genital opening (Figures 71, 72); length: 73 (71–83). Posterior limit epimere IV, oblique lineal ridge laterally directed (Figure 71, 72 indicated by ➧); originating in anterior zone of genital plate (Figures 71, 72 indicated by ➧). Complex structure lateral to setae *ag*, with a longitudinal cuticular elevation, parallel furrow and promontories (see leg folding “locking structure”); genital plate smaller than anal plate (Figures 71, 72).

Anal aperture more or less rectangular with rounded anterior and posterior zones; two pairs of anal setae (Figures 61, 71, 72), length: 37 (35–39); three pairs of adanal setae: *ad_1_*: 73 (71–75); *ad_2_*: 75 (73–78); *ad_3_*: 80 (77–83). Three pairs of subcapitular setae: *h, m* simple, barbate; *a* simple, smooth: lengths: *a*: 12 (9–15); *m*: 29 (28–33); *h*: 42 (39–45).

**Figures 73–76.**
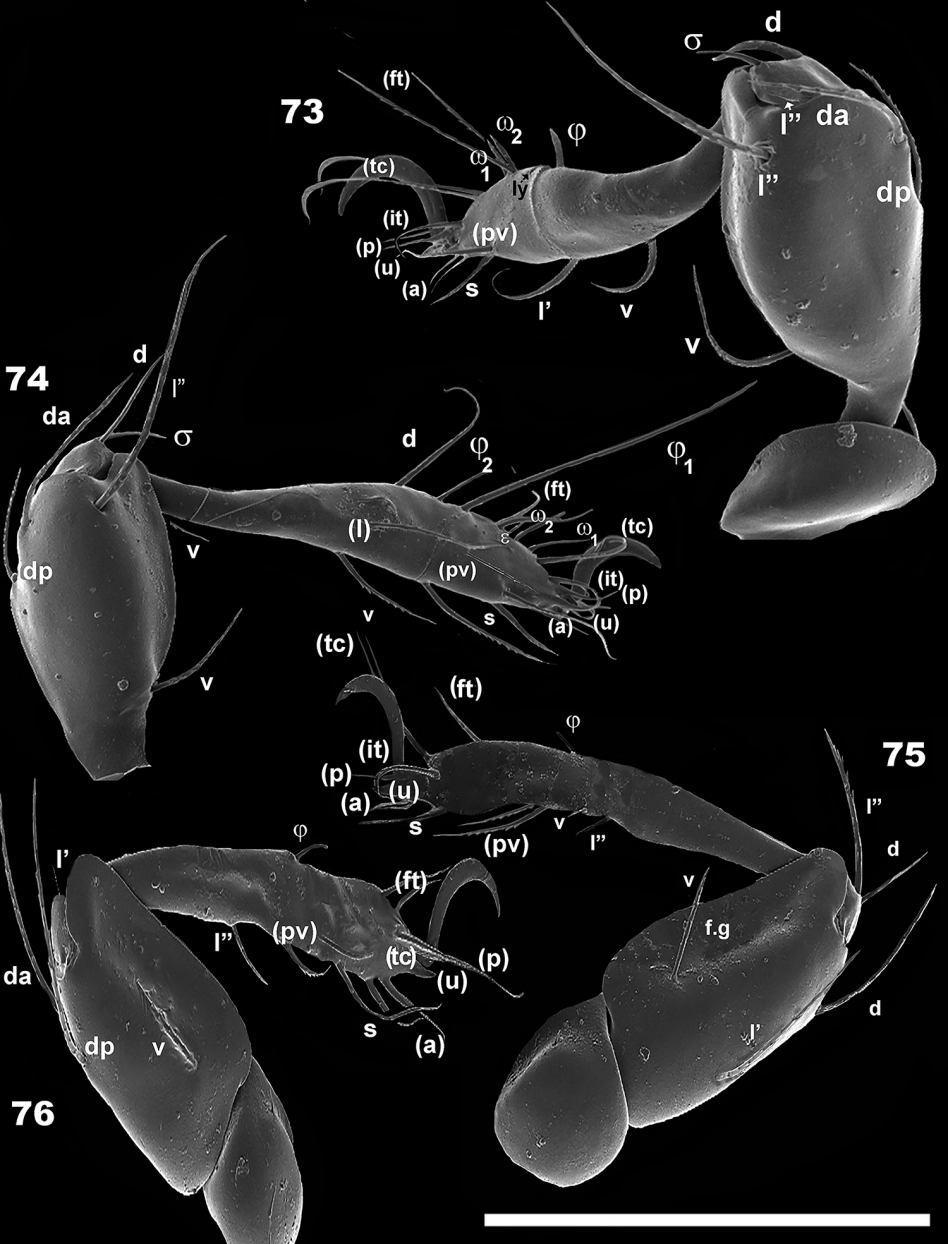
*Leobodestrypasis* sp. n. Adult female, SEM observations.**73**. leg II, antiaxial **74** leg I, antiaxial **75** leg III, antiaxial **76** leg IV, antiaxial. Scale bar: 130 μm (**73**–**76**).

***Legs*** (Figures 73–76, Table 2). Very small genua and long tibia in all legs. Femur III with *f.g* (Figure 75) (see Leg-folding process). Setal formulae: I (1-4-2-4-16-1) (1-2-2); II (1-4-3-2-15-1) (1-1-2); III (2-3-1-2-15-1) (0-1-0); IV (1-2-1-2-14-1) (0-1-0).

**Table 2.** *Leobodestrypasis* sp. n. setae and solenidia.

	Femur	Genu	Tibia	Tarsus	Claw
**Leg I**					
setae	*da,dp,l”,v*	*d,v*	*d,(l),v*	*(pv),(it),(tc),(ft),e, (p),s,(a) (u)*	1
solenidia		s	j_1_, j_2_	w_1_, w_2_	
**Leg II**					
setae	*da,dp,v,l*”	*d,l*”	*l’,v*	*(ft) (tc) (it) (p) (u) (a),s (pv)*	1
solenidia		s	j	w_1_ w_2_	
**Leg III**				)	
setae	*l’,v*	*l’,v,d*	*d,l*”	*(ft) (it) (tc)(p) (u) (a),s, (pv)*	1
solenidia			j		
**Leg IV**					
setae	*da,dp,v*	*l*’	*(l),v*	*(p) (u) (a) s, (pv) ft” (it) (tc)*	1
solenidia		-	j	-	

**Table 3.** Comparison of *N.panemorfis* sp. n. and *N.flagellifer* Chen & Wang, 2007.

	*Nippobodespanemorfis* sp. n.	*Nippobodesflagellifer* Chen & Wang, 2007
Rostrum	rounded, conspicuous parallel groove to margin; large hump in front of groove.	rostrum protruding dorsally
*p.p.d*.	deep, rounded-ovoid	polyhedral
Interlocking double hook	*h.ap* situated externally, grips on to *p.pr.co* on the interior	large rectangular *h.ap* interlocking with posterior part of *p.pr.co*, triangular to polyedral in shape (Fig. 18 Chen and Wang 2007)
*Tu*	strong, large lamina, anterior zone ending in sharp angle, with interior part welded to prodorsal wall	well developed, large lamina, blunt tip (Fig. 20 Chen and Wang 2007)
*n.a.d*	deep, rounded-ovoid	polyhedral
*c* setae	looped, dentate, sharply tipped	proximal half directing anteromedially, distal half curving posterolaterally
*h_3_, p_3_*	inserted on conspicuous promontories; v-shaped incision behind setal insertion	Neither promontories nor incision observed (Chen and Wang 2007: Fig. 18)
*s.c*	completely surrounding notogaster; originating slightly in front of *la* setae, running between *la, h_1_, h_2_* and *h_3_, p_3_, p_2_, p_1_*	starts behind *h*_3_ insertion, running between *h*_1_, *h*_2_ and *p*_1_, *p*_2_, *p*_3_ (Chen and Wang 2007: Fig. 18)

## Discussion

Aoki, when establishing the new genus *Nippobodes* in 1959, initially included it in the family Carabodidae. Almost sixty years later, we propose that the family Nippobodidae presents a series of characters linking these families, as knowledge of the families Nippobodidae and Carabodidae has grown significantly in the intervening years. We consider here only some elements that indicate important similarities: 1) prodorsal posterior depression and notogastral anterior depression situated either side of *d.sj*; 2) the projection of *h.ap* overlapping the posterior area of prodorsum; 3) the structures involved in leg folding such as tutorium, pedotectum I, genu (functioning as a hinge), femoral groove in femur III, shapes of femurs. These three elements are insufficient for a comparison, but highlight some aspects indicating a possible relationship between the families. More detailed analysis is required, but hampered by the lack of immature specimens of Nippobodidae and for the greater part of Carabodidae.

Unfortunately descriptions of genera in the family Nippobodidae are often superficial, and in many instances the frontal and posterior views were neglected although they could potentially provide important information. Leg chaetotaxy is problematic and we endeavour to obtain new material in order to study legs in a larger number of specimens. Much of our study material was collected many years ago, and does not permit detailed study, resulting in leg chaetotaxy necessarily being considered provisional.

It has been difficult to find a species related to *Nippobodespanemorfis* sp. n. due to its particular characteristics. *Nippobodesflagellifer* Chen & Wang, 2007 displays the most characters in common, as both species present similar disposition of: setae *ro* on tubercle near lateral margin of prodorsum; setae *le* inserted on tubercle on anterior on lamella; sensillus curved, sickle-shaped, swollen medially; posterior prodorsal condyles interlocking with notogastral humeral apophyse (but dissimilar in shape). Notogastral surface smooth; ten pairs of notogastral setae.

The taxonomy of *Leobodestrypasis* sp. n. is complex. The species is difficult to compare to other congeners due to their dissimilarity, and the often simplified original descriptions impede adequate comparison. However, there are similarities to *L.anulatus* Aoki, 1965, such as the presence of a heart-shaped prodorsal orifice, but occurring in a dorsal and not frontal position as in *L.trypasis*.

### *Leg-folding process* (Figures 77–88)

Fernandez et al. (2013a) have studied the folding of legs as a part of the protection mechanism in various genera of the family Carabodidae. We were fortunate to have the opportunity to examine this process in vivo on adults of *Carabodes* sp. under light microscopy and document the different steps. Additionally, material was available for SEM-studies, facilitating comparison with other SEM images. For this paper we were unable to conduct in vivo studies of the leg-folding process, but based on a series of observed morphological characteristics and a large number of SEM observations, we do not doubt the presence of similar functions and processes as observed in genera of the family Carabodidae.

Some morphological characteristics, however, suggest some variation in aspects of this mechanism.

To understand this process one needs, ﬁrst of all, to embark on detailed studies of leg structures in *Nippobodes* (Figures 48–53) and *Leobodes* (Figures 73–76), as well as other body structures related to leg positioning. Due to the high number of images obtained from more than fifty specimens, selected from a total of more than three hundred animals, only the most representative images of this mechanism have been included for the sake of clarity.

### Structures involved

The legs

**Figure 77–81.**
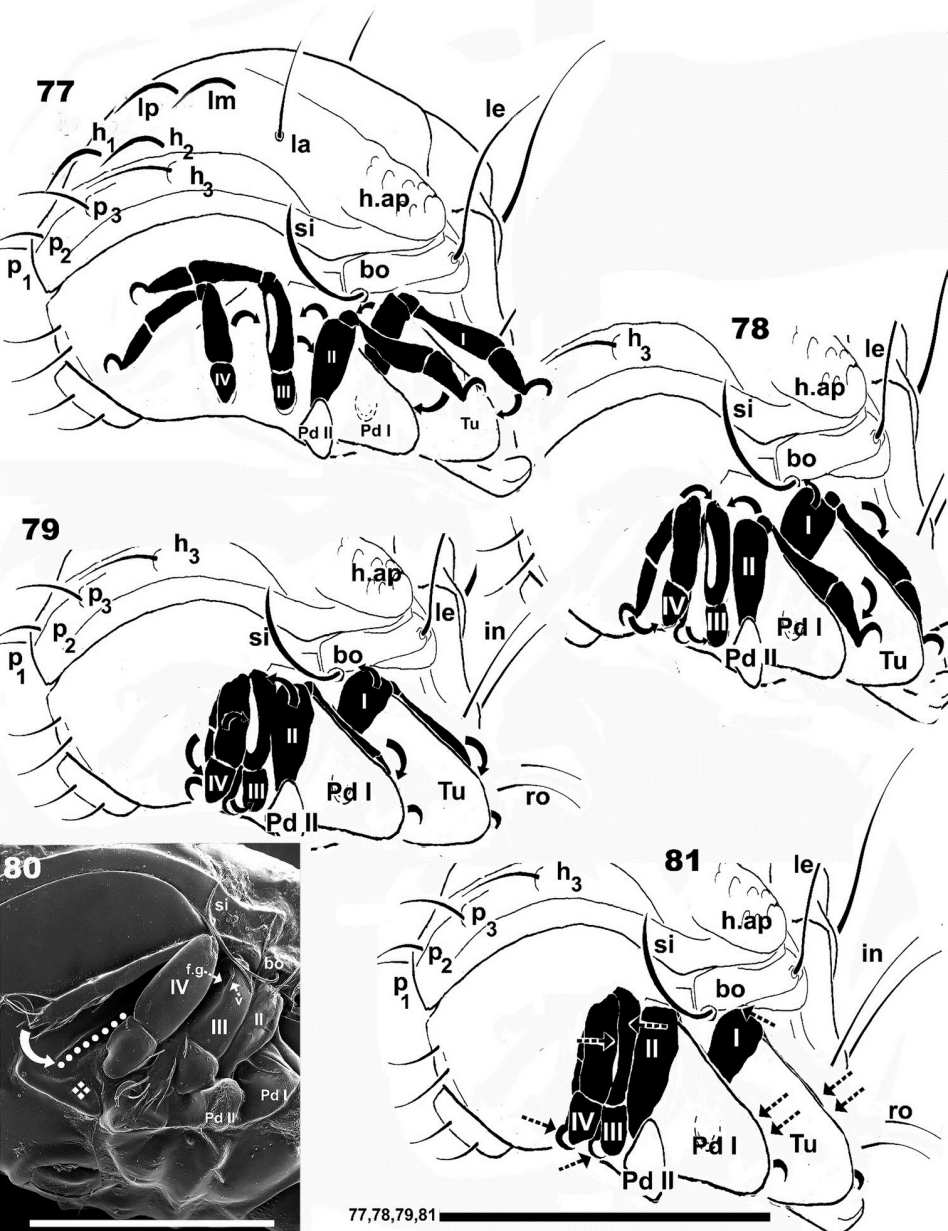
Schematic figures of leg-folding process based and SEM observations of *Nippobodespanemorfis* sp. n. **77** phase I **78** phase II **79** phase III **81** final phase **80** complementary explanation for insertion of tarsus and tibia IV into longitudinal depression. Scale bars: 390 μm (**77–81**); 300 μm (**80**).

**Figures 82–84.**
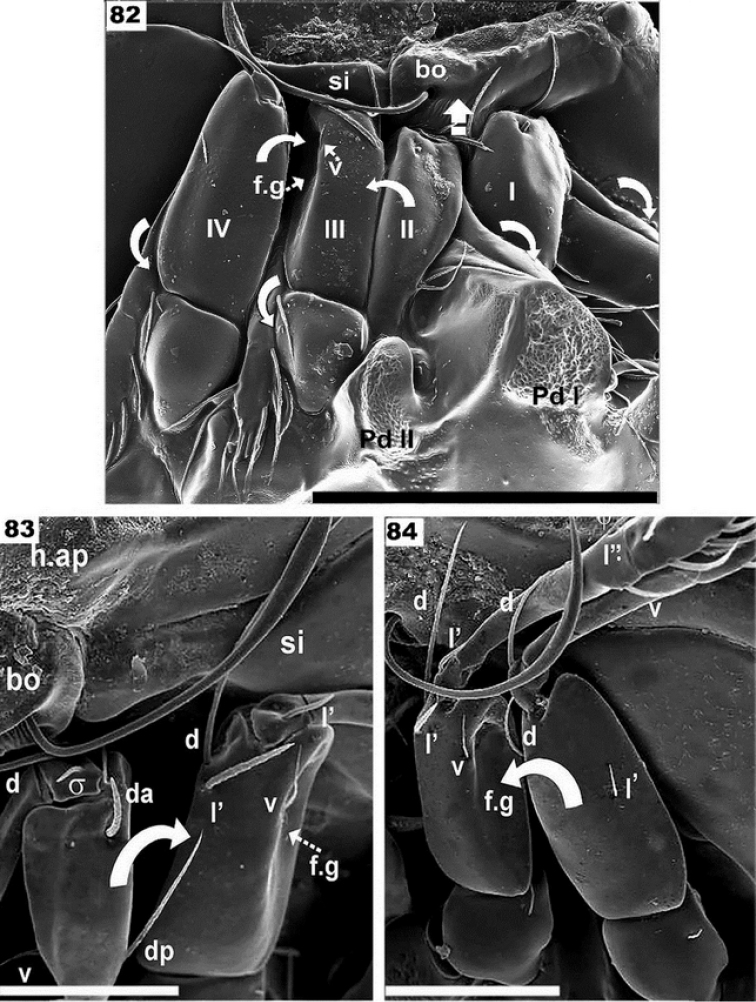
Complementary figures of leg-folding process. SEM observations of *Nippobodespanemorfis* sp. n. **82**. position close to the final stage of the leg-folding process **83** moment when the femur II approaches the back of the femur III **84** femur III and IV, femur III moving towards femoral groove. Scale bars: 200 μm (**82**); 100 μm (**83**, **84**).

**Figures 85–88.**
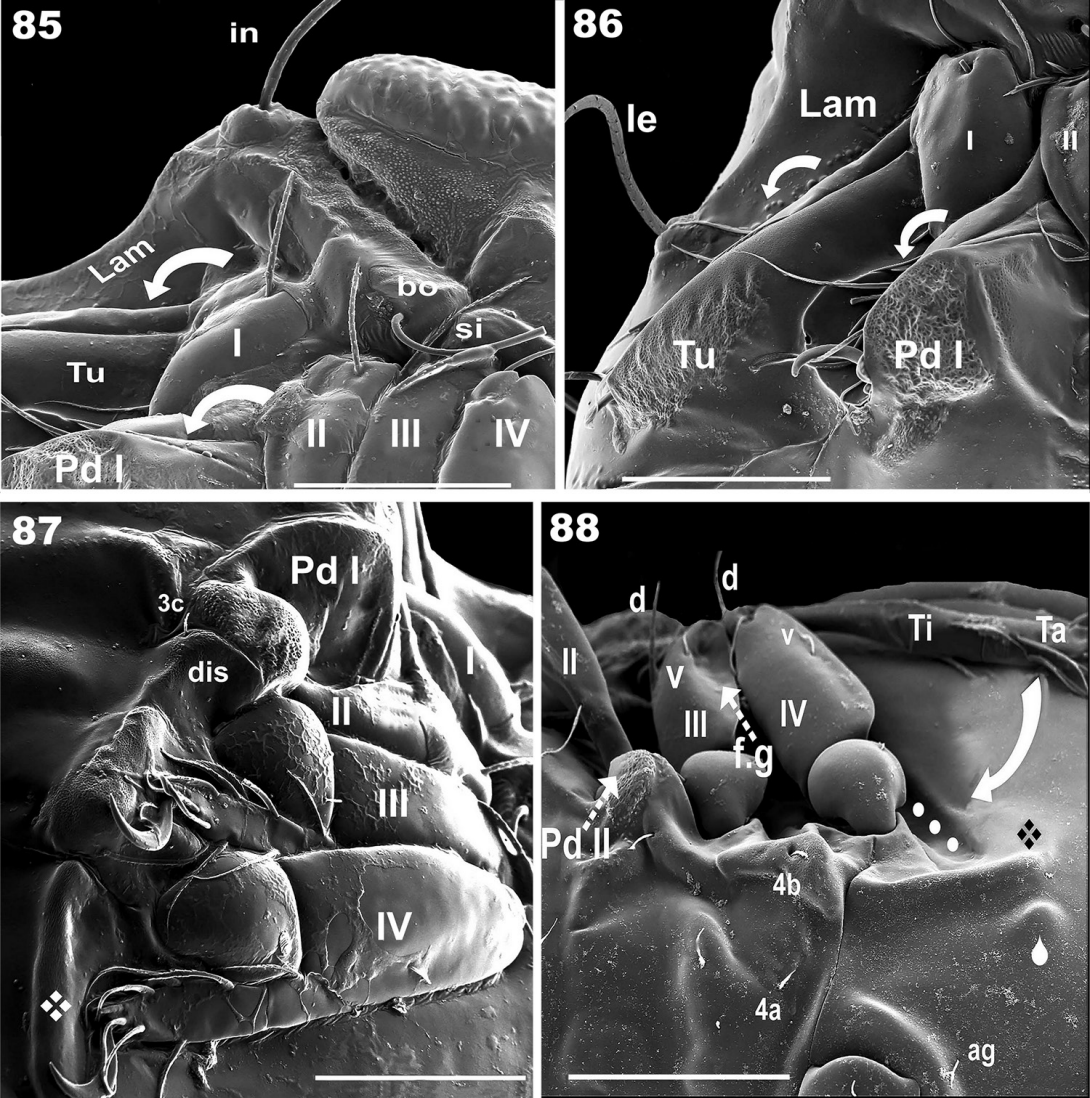
Complementary figures of leg-folding process. SEM observations of *Nippobodespanemorfis* sp. n. **85** lateral posterior–anterior view, final position of legs I and II **86** final position legs I and II **87** posterior–anterior view, final position of tarsus III and IV **88** legs III, IV and cuticular structures involved in leg folding, legs III and IV lateral view. Scale bars: 100 μm (**85**, **87**, **88**); 150 μm (**86**).

The following is generally observed: tibia-tarsal articulation by means of a small section of synarthrodial membrane, allowing limited movement. Tibia and tarsus are long and narrow, facilitating positioning either in a pocket-shaped structure delimited by the tutorium (See below), or behind pedotectum I, femur III, and IV (Figures 77–88). Particular characteristics present in superior part of femur I, allows for partial concealment under the lateral prodorsal zone, in front of the bothridial zone (Figure 82). Femur II presents a slightly curved, smooth posterior surface (Figure 83), coapting with the posterior area of femur III. These structures and surfaces of femurs III and IV permit perfect coaptation, to allow tibia and tarsus to slip in behind and be concealed by them (Figures 82, 87).

Leg III plays a vital role. The femoral groove on femur III is a rather deep, triangular to ovoid groove, with a small seta near the depression. The groove and seta permit anchoring of femur IV into the groove. Femora III and IV each presents a ventral carina, permitting the tibia and tarsus to be concealed under them (Figures 75, 76, 82, 84).

The tiny genu plays a fundamental role as hinge, and generally presents a reduced number of setae (Figures 48–53; 73–76). The particular shape of the anterior zone of the femur improves the genu-hinge function and assists in the tibia-tarsus in the required position.

### Related structures

*Tutorium*

The tutorium plays a very particular role, forming a pocket-shaped structure with the lateral prodorsal zone, permitting concealment of tibia-tarsus I. The pocket shaped structure ends in a sharp point, which protects the leg, and houses the claw (Figures 56, 57). Pedotectum I conceals the tibia and tarsus of leg II.

*Lateral zone of body*

The lateral area of body is adapted to receive the legs, with depressions and smooth areas to facilitate their positioning, along with tutorium, pedotectum I, and between legs II, III, and IV.

*The “locking structure*”

Ventrally, behind leg IV, a locking structure is observed. It consists of a longitudinal furrow (on Figures 42, 62, 80, 88, trajectory indicated by ●), ending in a bean-shaped structure (on Figures 42, 80, 87, 88 indicated by ❖), with a lateral promontory (Figures 42, 88, indicated by 🌢). Tibia-tarsus IV is inserted into the longitudinal furrow (Figure 80) and the claw positioned in the depression of the bean-shaped structure (Figures 80, 87, 88). The femur resembles a lid closing a box, preventing the tibia-tarsus from moving and anchoring the entire leg in one position (Figure 81, 82, 88).

### The process

*Phase 1* (Figure 77): Initial position prior to leg folding, arrows indicating the directions in which legs will move.

*Phase 2* (Figure 78): Leg I: femur moves backwards and approaches bothridial zone; this movement is facilitated by the genu functioning as a hinge. By rotating, it permits the tibia and tarsus to approach the margin of tutorium. Then, the tibia and tarsus are positioned ready to initiate installation into the tutorium pocket. Leg II: the femur moves upward and backward, approaching the posterior zone of femur III; the tibia and tarsus approach the margin of pedotectum I and move downwards, to conceal those two segments behind pedotectum I. Leg III: rotates towards the posterior and femur III moves closer to femur II. The tibia and tarsus slide in under ventral trochanteric-femoral carinas for concealment. Leg IV: the femur is directed backwards in order to locate the femoral groove and settle into it. The tibia and tarsus move back to settle into the longitudinal depression indicated by ● (Figure 80).

*Phase 3* (Figure 79): Leg I. Femur I approaches the final position on the bothridial zone; the tarsus and tibia are almost completely concealed behind the tutorium and embedded in the pocket tutorial depression. Leg II. Femur is coapted to the posterior zone of femur III. The tibia and tarsus are almost completely hidden behind pedotectum I. Legs III and IV are very close to each other; femur IV is almost entirely within the femoral groove of femur III. Tibia and tarsus IV are installed in the longitudinal depression of the locking structure and tibia and tarsus III slide in and are concealed under femoral and trochanteral carinae; the claw is visible between trochanters III and IV.

*Phase 4, the ﬁnal position* (Figure 81). Leg I: apical dorsal area of femur positioned under the anterior part of bothridium (indicated by 3) . The genu rotates inwards and its dorsal part, as well as the tibia and the tarsus are concealed deep in the tutorial pocket depression. Leg II. The genu turns inwards to position the tibia and tarsus in the optimal position; the tibia is slightly curved. With slight rotation of the genu, the tibia is similar in shape to pedotectum I; the tibio-tarsus articulation gives important rigidity. The tibia and tarsus descend and are perfectly concealed behind pedotectum I. Leg III. The posterior zone of femur II glides underneath the anterior part of femur III. Tibia and tarsus III slide in under the carina of trochanter–femur III for concealment, and the tarsal claw is visible between trochanters III and IV. Leg IV: the posterior part of femur IV is placed inside the femoral groove of femur III. Femur IV is inclined upwards to enable the tibia and tarsus to glide in underneath the femur, and tibia and tarsus IV are placed into the longitudinal furrow of the locking structure. To conclude the mechanism, femur IV acts as a lid, blocking this segment and concealing the leg segments in the longitudinal depression, with the claw placed in a horizontal position resting on the bean-shaped structure of the locking structure (Figure 80).

During the ﬁnal stages of the coaptation process, the relationship between the legs and body depressions can be described as follows: tibia and tarsus IV are located in the longitudinal furrow (of the locking structure), concealed by the femur; the posterior part of femur IV anchors in the groove of femur III; the apical distal expansion of femora III and IV partially conceal the genu; apical zone of tarsus III is situated between trochanter III and trochanter IV (Figures 79, 81). The *f.g* allows the posterior part of femur IV to fold into femur III.

### Supplementary SEM images

In Figure 82 the final position has almost been reached, with an indication of structures involved, and arrows indicating the final movement to conclude the process. Figure 83 indicates the step where femur II is directed to the anterior part of femur III. The surfaces of the femurs about to come into contact can clearly be seen to fit together perfectly, and other structures such as the setae are located in such a way that they do not impede this process. On the posterior zone of Femur III, the femoral groove shows the seta *v*, which will assist in anchoring femur IV inside the depression. Figure 84 shows the displacement of femur IV towards the femoral groove (femur III). The carina of the trochanter and femur is clearly visible, which will permit the tarsus and tibia of leg III and leg IV to be partially concealed. Figure 85 posterior view, indicates the final position of legs I, II. The arrows indicate the position of tibia and tarsus I and II, concealed behind the pedotectum I and tutorium. Figure 86 Shows the lateral view of final position of legs I and II. Figure 87, posterior view. Tarsus III and tarsus IV are clearly visible, as well as the claw of tarsus IV and the bean-shaped structure of the locking structure supporting claw IV. Figure 88 shows theset of fundamental elements of legs III and IV, as well as cuticular surfaces necessary for the leg-folding process.

*Similarities and differences in leg folding between Carabodidae and Nippobodidae*

The system is very similar in the two families, and importantly, the following are common to both: all legs are involved in the process; the presence of the femoral groove on femur III; a tiny genu, which plays the role of a hinge; the involvement of pedotecum I and tutorium to conceal legs I and II.

Differences: 1) In Nippobodidae leg I is concealed in a pocket structure formed by the attachment of the tutorium to the lateral wall of the prodorsum. This connection to the prodorsal body wall resulting in the formation of the pocket structure is very different to Carabodidae (see Fernandez et al. 2013a). 2) The complexity of the locking structure in Nippobodidae, specifically the longitudinal depression where tibia and tarsus IV are inserted, femur IV which functions as a lid, and the bean-shaped structure where the claw rests, are dissimilar to what is observed in Carabodidae.

## Supplementary Material

XML Treatment for Nippobodes panemorfis

XML Treatment for Leobodes trypasis
